# 仪器分析实验设计与实践：多次顶空萃取-气相色谱-质谱测定野菊花中的龙脑和樟脑

**DOI:** 10.3724/SP.J.1123.2025.09030

**Published:** 2026-07-08

**Authors:** Huixiang LI, Wentao JIANG, Lihang ZHOU, Han ZHENG, Shasha LIU, Jie LEI

**Affiliations:** 复旦大学化学系，上海 200433; Department of Chemistry，Fudan University，Shanghai 200433，China

**Keywords:** 多次顶空萃取, 气相色谱-质谱, 樟脑, 龙脑, 野菊花, 实验教学, multiple headspace extraction （MHE）, gas chromatography-mass spectrometry （GC-MS）, camphor, borneol, *Chrysanthemum*, experimental teaching

## Abstract

本研究在原有实验及科研课题基础上，通过拓展仪器功能设计了一个本科仪器分析实验，旨在引导师生深入理解并重视样品前处理技术在分析流程中的关键作用。实验以野菊花为分析对象，建立了一种基于多次顶空萃取-气相色谱-质谱（MHE-GC-MS）联用测定其中樟脑与龙脑含量的分析方法。通过系统优化顶空平衡温度与平衡时间等关键参数，确定最佳前处理条件为顶空平衡温度100 ℃、平衡时间20 min。方法学验证表明，目标化合物具有良好的线性关系，相关系数（*R* ^2^）为0.981 2~0.999 2。应用所建立的方法测得野菊花中樟脑与龙脑的含量分别为0.917 4 mg/g与0.944 2 mg/g，结果显著高于传统水蒸馏-溶剂萃取法，体现了MHE技术近乎完全萃取的优势。本实验作为一项综合探究性教学项目，成功融入仪器分析实验课程，有效培养了学生在方法开发、条件优化与数据处理等方面的综合实践能力与科学思维。本研究提出的教学方法无需增加实验试剂等，具有较好的普适性、可拓展性和推广价值，可为仪器分析实验教学改革提供有益参考。

## Abstract (en)

On the basis of previous experiments and research projects， this study developed an undergraduate instrumental analysis experiment by expanding the functionality of existing instruments. The primary objective was to enhance awareness among both instructors and students regarding the importance of sample pretreatment. Using *Chrysanthemum* as the analytical sample， an analytical method for determining the content of camphor and borneol was established based on multiple headspace extraction-gas chromatography-mass spectrometry （MHE-GC-MS）. By systematically optimizing key parameters such as headspace equilibration temperature and time， the optimal pretreatment conditions were determined as an equilibration temperature of 100 ℃ and an equilibration time of 20 min. Method validation demonstrated that both the liquid and solid external standard methods exhibit good linear relationships， correlation coefficients （*R*²） ranging from 0.981 2 to 0.999 2. Using the established method， the measured contents of camphor and borneol in *Chrysanthemum* were 0.917 4 mg/g and 0.944 2 mg/g， respectively. These results are significantly higher than those obtained by traditional hydrodistillation-solvent extraction， reflecting the near-complete extraction advantage of MHE. As a comprehensive inquiry-based teaching project， this experiment has been successfully integrated into the instrumental analysis laboratory course， effectively cultivating students’ comprehensive practical skills and scientific thinking in method development， condition optimization， and data processing. The proposed teaching method does not require additional experimental reagents and demonstrates good universality， extendibility， and promotability， providing valuable insights for the reform of instrumental analysis laboratory teaching.

仪器分析实验是化学及相关专业的核心课程^［1］^，实验内容涵盖色谱分析、光谱分析、电化学分析等多种方法^［2-7］^。样品前处理是仪器分析实验的关键步骤，直接影响分析结果的准确性和可靠性，其目的是去除干扰物质，同时提高目标物的浓度，使其适合仪器检测。例如，色谱分析前需去除颗粒物，光谱分析前需溶解或稀释样品。合理的前处理不仅能提高方法选择性、灵敏度和准确度，还能延长仪器使用寿命^［8］^。

在当前分析实验室面临样品通量持续增长的背景下，自动化就变得越来越重要，而顶空-气相色谱联用技术（HS-GC）就是一个理想的选择。HS-GC是将样品（固体或者液体）置于密闭容器中，通过加热样品使其中的挥发性组分进入样品上方的气相，再通过顶空进样器将挥发性组分直接引入气相色谱仪进行分析。这一技术结合了自动化与样品前处理的优势：自动化顶空进样器可实现样品的批量处理，减少人为误差，提高实验效率；顶空技术本身是一种无需复杂前处理的样品制备方法，避免了溶剂干扰和样品损失，特别适用于复杂基质（如食品、环境样品）中挥发性成分的分析^［9］^。

顶空分析（HSA）主要可以分为静态顶空分析和动态顶空分析，除此之外，还有一些基于顶空原理的辅助技术，比如顶空-固相微萃取、多次顶空萃取等。多次顶空萃取（MHE），又称为多级顶空萃取，是一种特殊的静态顶空技术，通过连续多次地对同一样品进行顶空取样和分析，直至顶空中不再能检测到目标物为止。MHE方法和数学模型最初由McAuliffe和Suzuki等提出，最初的MHE步骤中，第二次顶空分析前去除了与样品相平衡的整个气相部分，后来Kolb发现只需要除去部分顶空相即可，因为在下一次分析前，两相之间已再次建立新的平衡^［9］^。理论上，各次萃取所测得的目标物含量之和应等于原始样品中该组分的总量。在实际操作中，通常不进行无限次萃取，而是基于有限次数连续萃取所获得的峰面积数据，通过数学模型推算出样品中待分析物的初始总量。目前用于本科实验教学的HSA主要有固相微萃取^［10-13］^和静态顶空^［14］^，未见多次顶空萃取的报道。

本实验在前期工作^［15-18］^的基础上，将多次顶空萃取-气相色谱-质谱联用技术用于野菊花中龙脑和樟脑的测定，让学生系统优化顶空平衡温度与平衡时间等关键参数，并将其所得实验结果与传统水蒸馏-溶剂萃取法^［16］^进行比较，同时还鼓励学生更换测量体系，有效培养了学生在方法开发、条件优化与数据处理等方面的综合实践能力与科学思维，并使学生充分认识到样品前处理的重要性及其多样性。

## 1 实验部分

### 1.1 仪器、试剂与材料

BP221S电子天平（赛多利斯，德国）；7697A顶空进样-7890B气相色谱-5977A质谱仪（安捷伦，美国），多功能小型粉碎机（金富瓷器有限公司）；150 mm玛瑙研钵（上海精密仪器仪表有限公司）。

野菊花样品（福曦堂），樟脑标准品、龙脑标准品（>98.0%，东京化成工业株式会社），无水硫酸镁（分析纯，上海大合化学品有限公司），乙酸乙酯（分析纯，国药集团化学试剂有限公司）。20 mL顶空瓶（安捷伦，美国）。

### 1.2 分析条件

#### 1.2.1 顶空条件

平衡温度100 ℃，环线温度110 ℃，传输线温度120 ℃，平衡时间20 min，进样时间0.5 min，样品压力103 421 Pa。每个样品连续进样5次，每次采样后，部分顶空被放空，以使顶空瓶的压力恢复到大气压。

#### 1.2.2 气相色谱条件

HP-5 MS石英毛细管色谱柱（30 m×0.25 mm×0.25 μm，美国J&W Scientific公司），载气为氦气，流速1.0 mL/min；进样口温度250 ℃，分流比50∶1；程序升温如下：60 ℃保持1 min，以12 ℃/min升温至160 ℃，保持1 min，再以30 ℃/min升温至280 ℃，保持3 min，总过程17.33 min。

#### 1.2.3 质谱条件

电离方式：电子轰击电离（EI，70 eV）；离子源温度230 ℃；四极杆温度150 ℃；溶剂延迟4 min；采用全扫描（SCAN）检测模式，质量扫描范围*m*/*z* 35~500；由Chemstation工作站软件采集数据，由NIST 11数据库对检测到的化合物进行谱库检索和比对。

### 1.3 标准样品配制及试样处理

#### 1.3.1 液体标准样品的配制

称取樟脑20 mg、龙脑10 mg，用乙酸乙酯溶解并定量转移至50 mL容量瓶中，定容，得到樟脑、龙脑标准溶液。分别移取樟脑、龙脑的标准溶液各0、0.5、1.0、1.5、2.0、2.5 mL于6个5 mL容量瓶中，用乙酸乙酯定容，得系列标准溶液。用50 μL的微量注射器分别取50 μL系列标准溶液，转移至6个20 mL顶空瓶，立即封盖，待分析。

#### 1.3.2 固体标准样品的配制

准确称量樟脑20 mg、龙脑15 mg、无水硫酸镁10 g，转移至玛瑙研钵，研磨5 min；从该混合物中准确称取0.4 g，再加入1.6 g无水硫酸镁，研磨5 min^［18］^。准确称量上述固体标准样品10、20、40、60、80、100 mg，转移至6个20 mL顶空瓶中，立即封盖，待分析。

#### 1.3.3 试样处理

准确称取野菊花10 g，用粉碎机粉碎，取粉碎后试样50 mg 6份，置于6个20 mL顶空瓶中，立即封盖，待分析。

## 2 结果与讨论

### 2.1 实验条件优化

#### 2.1.1 色谱条件

质谱分析条件的优化步骤详见相关文献^［15，16］^。为了实现野菊花中的龙脑和樟脑与其余成分的基线分离，尝试了不同的气相色谱程序升温条件，得到实验部分所述气相色谱条件即为最佳实验条件。图1是最佳实验条件下的典型色谱图。

**图1:**
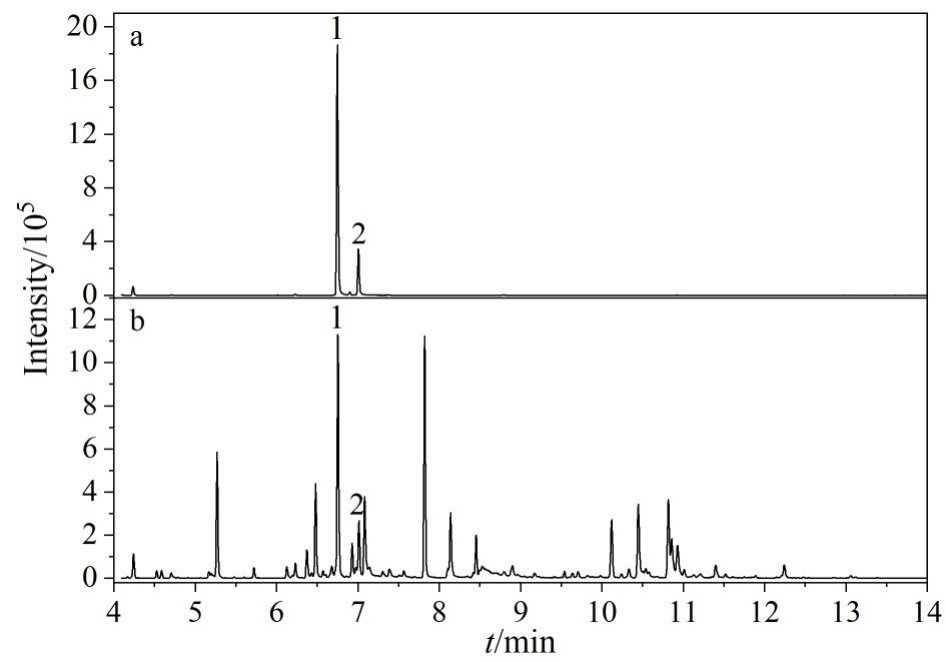
（a）固体标准试样和（b）野菊花样品的总离子流色谱图

#### 2.1.2 顶空平衡温度

顶空平衡温度是影响野菊花样品中樟脑与龙脑测定结果的关键因素。在固定顶空平衡时间为20 min的条件下，系统考察了不同平衡温度对樟脑和龙脑含量的影响，结果如图2所示。随着温度升高，两种组分的含量均呈现上升趋势，尤其在95~100 ℃区间内增幅最为显著。在95 ℃及以下温度时，顶空瓶内气-液/固两相未能充分达到平衡，导致樟脑与龙脑的测量值偏低；而当温度升至100 ℃时，系统趋于平衡状态。在实验所采用的直接顶空进样系统中，传输线的最高耐受温度为120 ℃。根据仪器要求，环线温度需低于传输线温度10 ℃，而顶空瓶的平衡温度需进一步低于环线温度10 ℃。基于该仪器限制与相平衡行为，最终确定100 ℃为适宜的顶空平衡温度。

**图2:**
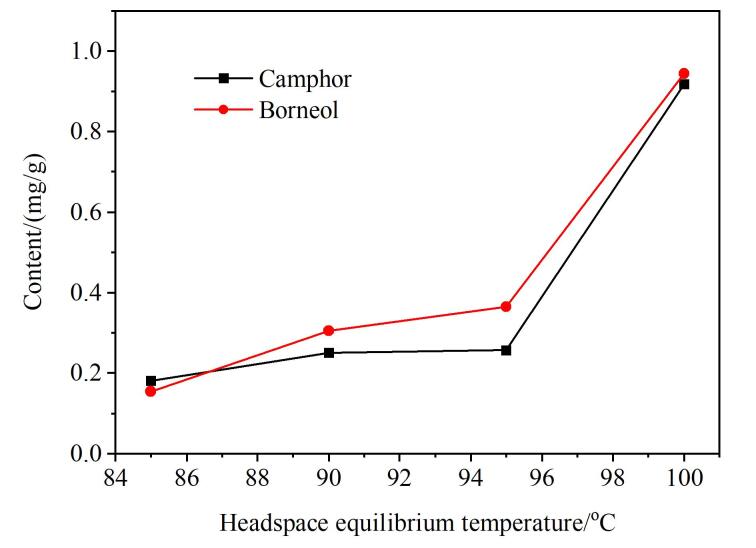
不同顶空平衡温度下野菊花中樟脑、龙脑的含量

#### 2.1.3 顶空平衡时间

在平衡温度保持100 ℃的条件下，考察了不同平衡时间对测定结果的影响，结果如图3所示。随着平衡时间的延长，野菊花中樟脑和龙脑的测定含量逐渐上升，并在30 min时达到最大值，表明30 min为该实验条件下的理想平衡时间。然而，20 min的平衡时间同样具有实际应用价值。一方面，在该时间点已能检测出样品中大部分的樟脑与龙脑；另一方面，实验所采用气相色谱方法的单次分析周期（包括进样与仪器降温过程）总计约20 min，与20 min的平衡时间基本匹配，可实现样品连续平衡与进样的无缝衔接，有助于提高仪器使用效率并缩短整体分析时间。因此，最终确定20 min为优化的顶空平衡时间。对于低含量的其他化合物，为了提高灵敏度和准确性，建议以含量最大值时的时间为最佳顶空平衡时间。

**图3:**
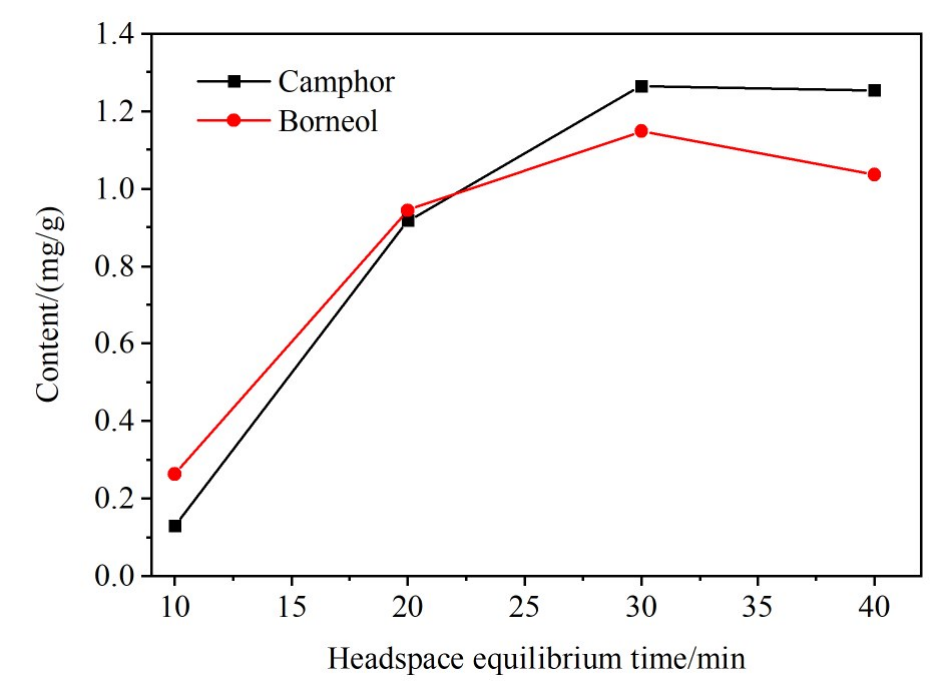
不同顶空平衡时间下野菊花中樟脑和龙脑的含量

### 2.2 多次顶空方法学考察

在多次顶空萃取中，若从同一顶空瓶连续两次提取相同体积的顶空气体，第二次进样的色谱峰面积通常小于第一次。随着萃取次数的增加，顶空瓶中待测组分的含量逐渐降低，其相应峰面积呈指数衰减，直至该组分被完全萃取（图4）。实验中，连续萃取得到待分析物的峰面积是一个收敛的几何级数，其总和见式（1） ^［9］^：


$$\sum_{i=1}^{i\to \infty }A_{i}=\frac{A_{1}}{1-e^{-q}}$$ （1）

其中，*A*_1_为第一次提取时获得的峰面积，*A_i_* 为第*i*次萃取时的峰面积，指数*q*为描述MHE过程中峰面积呈指数下降的指标，*i*为萃取次数。

**图4:**
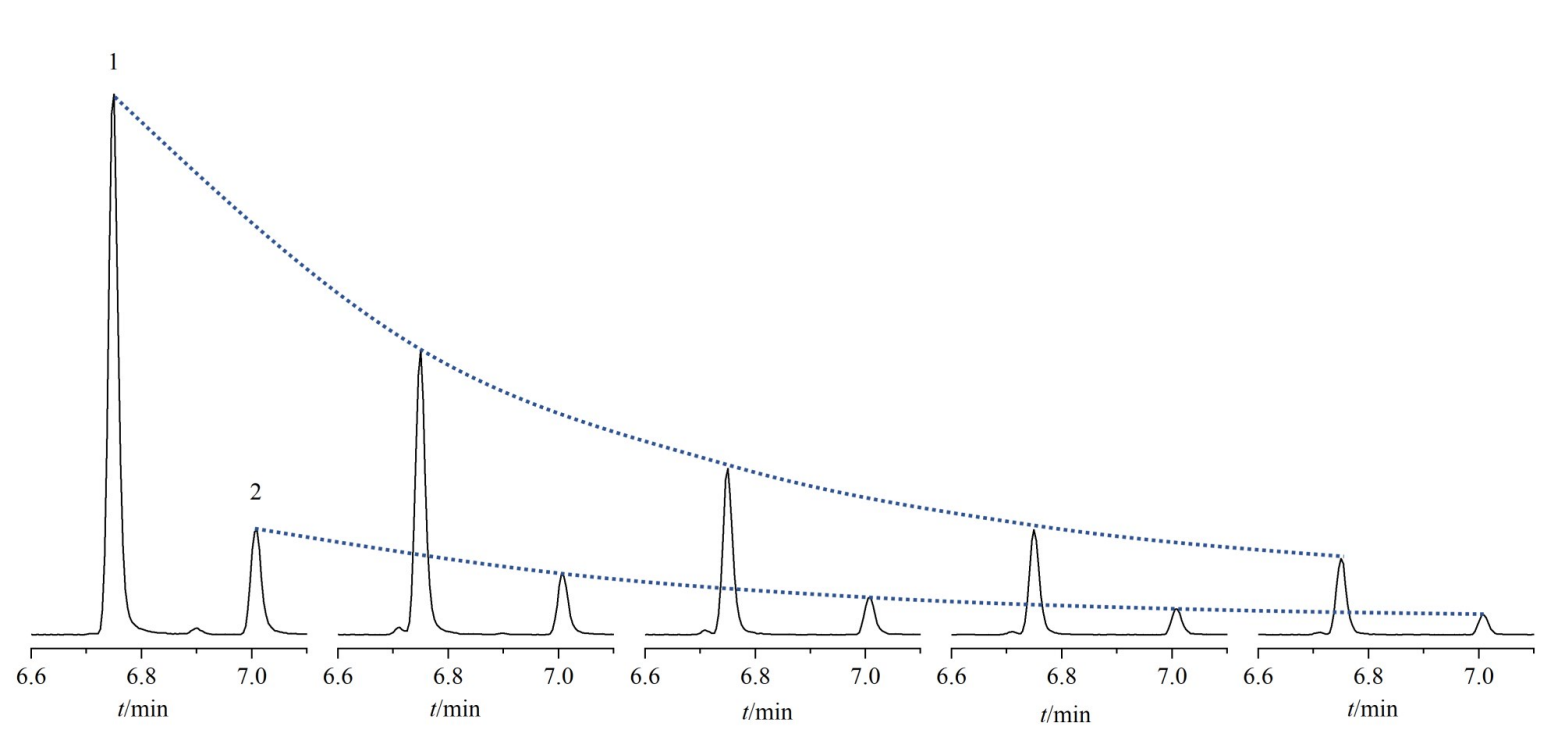
固体标准试样5次连续顶空萃取分析的总离子流色谱图

将气体连续提取的一阶机理进一步计算见式（2） ^［9］^：


$$ln\;A_{i}=-q(i-1)+ln\;A_{1}$$ （2）

这是一个*y*=*ax*+*b*类型的线性方程，其中*x*=*i*-1；斜率为-*q*，y轴截距是ln *A*_1_。

因此，在MHE中，将连续萃取得到的峰面积按照式（2）进行处理和线性回归（图5），可以求得*q*以及ln *A*_1_，代入式（1）计算可得总峰面积。将总峰面积与樟脑和龙脑的质量做标准曲线，可得表1。

**图5:**
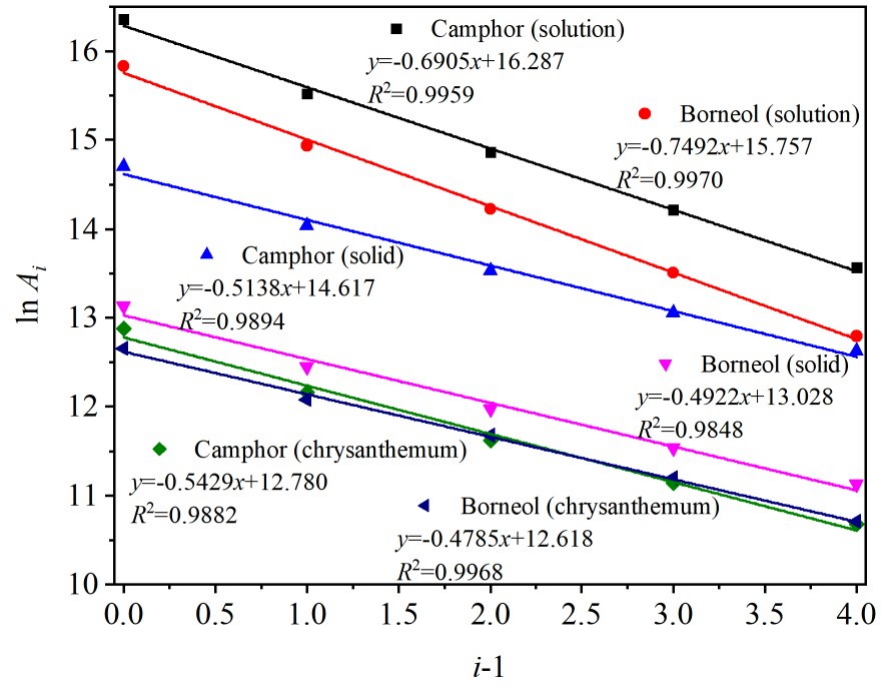
不同基质中2种化合物的线性回归曲线

**表1:** 樟脑和龙脑的线性方程、线性范围和相关系数

Compound	Linear equation	Linear range/μg	*R* ^2^
Camphor （solid）	*y*=1.2700×10^8^ *x*+556141	4-40	0.9958
Borneol （solid）	*y*=3.3874×10^8^ *x*+229415	3-30	0.9812
Camphor （solution）	*y*=1.2869×10^9^ *x*+91713	4-20	0.9992
Borneol （solution）	*y*=1.4299×10^9^ *x*-173556	2-10	0.9992

*y*： total peak area of compound； *x*： mass， μg.

对比4条标准曲线可知，在液体外标法中，樟脑和龙脑的质量与响应值之间具有更好的线性关系，其标准曲线的线性优于固体外标法所得结果。这可能是由于液体样品在顶空瓶中的挥发更为充分，使得气相中的待测物分布更均匀，从而提高了检测数据回归方程的相关性。

### 2.3 实际样品分析

在相同实验条件下对粉碎后的野菊花样品进行多次顶空萃取，将所得总峰面积代入上述固体标准曲线，计算得出样品中樟脑和龙脑的含量。结果显示，福曦堂野菊花中樟脑含量为0.917 4 mg/g，龙脑含量为0.944 2 mg/g，均显著高于文献中采用水蒸馏结合溶剂萃取法所报道的数值^［15，16］^。这一差异可归因于多次顶空萃取作为一种近乎完全的提取方式，能够更加充分地释放目标组分；而传统水蒸馏-溶剂萃取以及微波辅助等方法在加热和萃取过程中可能导致樟脑与龙脑的挥发损失或提取不完全，从而使测定值偏低。因此，本研究观测到的含量更高具有合理的理论依据。

## 3 实验的组织与教学安排

本实验已在“探究性仪器分析”课程中成功开展两轮完整教学实践。在教学实施过程中，学生系统性地参与了从文献调研、实验方案设计、分析方法建立与优化，到实际样品测定、数据整理与分析，直至最终撰写研究报告的全过程，全面培养了学生的科研素养与综合实践能力。此外，该实验也作为一项选修模块，在“仪器分析实验”课程中开设一轮。在该轮次中，学生主要聚焦于实际样品的测试环节，以及后续的数据分析与报告撰写，侧重于对仪器操作与数据处理能力的训练。

由于理论课并未讲授MHE的基本理论，所以在实验讲解过程中需重点讲授MHE所用公式及计算过程。考虑到多次顶空萃取-气相色谱-质谱联用仪器具备方法编程与自动序列进样功能，本实验可高效适配“6+2”课时安排：前6课时用于样品前处理、仪器方法与序列参数设定，随后启动仪器自动过夜运行；后续2课时则集中进行数据处理、结果分析与报告撰写。若作为探究性实验项目，可在此基础上增设约4~8课时，用于引导学生开展文献研读、研究方案设计与小组讨论，并可拓展样品类型，不局限于野菊花；另外，方法学验证部分还可参照文献［^19^］进行检出限、定量限、三水平加标回收试验和重复性考察等，以进一步强化学生的科学探究与创新思维能力。

对于尚未配备专用MHE-GC-MS系统的院校，本实验方案亦具备良好的可移植性。可采用常规加热板对顶空瓶进行空气浴或油浴加热，模拟顶空萃取过程，随后通过手动进样方式将顶空气体注入气相色谱系统进行分析。检测器可根据实际配置灵活选择，如氢火焰离子化检测器等，不一定依赖质谱，体现了教学方案的良好普适性与设备兼容性。

## 4 结语

本实验成功构建了一套基于多次顶空萃取-气相色谱-质谱联用技术的本科教学方案，实现了对野菊花中樟脑与龙脑的准确测定。该方案不仅系统性地传授了现代仪器分析的原理与操作技能，更通过“探究性”与“常规性”双轨并行的教学设计，引导学生完整经历从文献研读到报告撰写的全科研流程，有效培养了学生的综合实践能力与科学思维。

实验设计的突出优势在于其普适性。方案充分考虑了不同高校的硬件差异，提供了从全自动MHE-GC-MS到简易顶空-气相色谱的多元化实施路径，确保了教学内容的广泛适用。教学实践表明，学生最大的收获在于能够基于现有仪器平台，灵活运用所学化学原理进行方法开发与优化，这一过程极大地激发了学生的科研兴趣与创新自信。

展望未来，本实验体系具备显著的可拓展性。其方法学可迁移至多种实际分析场景，例如咖啡中咖啡因的快速测定或聚合物材料中残留单体的分析等。这种面向实际应用的延伸，将进一步训练学生的知识迁移与解决复杂问题的能力，为培养具备创新精神与分析素养的高素质化学人才提供坚实支撑。
